# Electrocardiogram Signal Analysis With a Machine Learning Model Predicts the Presence of Pulmonary Embolism With Accuracy Dependent on Embolism Burden

**DOI:** 10.1016/j.mcpdig.2024.03.009

**Published:** 2024-05-24

**Authors:** Waldemar E. Wysokinski, Ryan A. Meverden, Francisco Lopez-Jimenez, David M. Harmon, Betsy J. Medina Inojosa, Abraham Baez Suarez, Kan Liu, Jose R. Medina Inojosa, Ana I. Casanegra, Robert D. McBane, Damon E. Houghton

**Affiliations:** Department of Cardiovascular Medicine, Mayo Clinic, Rochester, MN

## Abstract

**Objective:**

To develop an artificial intelligence deep neural network (AI-DNN) algorithm to analyze 12-lead electrocardiogram (ECG) for detection of acute pulmonary embolism (PE) and PE categories.

**Patients and Methods:**

A cohort of patients seen between January 1, 1999, and December 31, 2020, from across the Mayo Clinic Enterprise with computed tomography pulmonary angiogram (CTPA) and ECG performed ±6 hours was identified. Natural language processing algorithms were applied to radiology reports to determine the diagnosis of acute PE, acute right ventricular strain pulmonary embolism (RVSPE), saddle pulmonary embolism (SADPE), or no PE. Diagnostic performance parameters of the AI-DNN reported were area under the receiver operating characteristics curve (AUROC), sensitivity, specificity, positive predictive value (PPV), and negative predictive value (NPV).

**Results:**

A cohort of patients with CTPA report and ECG consisted of 79,894 patients including 7423 (9.3%) with acute PE, among whom 1138 patients had RVSPE or SADPE. Artificial intelligence deep neural network predicted acute PE with a modest accuracy of AUROC of 0.69 (95% CI, 0.68-0.71), sensitivity of 63.5%, specificity of 64.7%, PPV of 15.6%, and NPV of 94.5%. The AI-DNN prediction using the same algorithm for RVSPE or SADPE was higher (AUROC, 0.84; 95% CI, 0.81-0.86) with a sensitivity of 80.8%, specificity of 64.7.8%, PPV of 3.5%, and NPV of 99.5%.

**Conclusion:**

An AI-based analysis of 12-lead ECG shows modest detection power for acute PE in patients who underwent CTPA, with higher accuracy for high-risk PE. Moreover, with the high NPV, it has the clinical potential to exclude high-risk PE quickly and correctly.

Of 120 million patients who visited emergency departments between 2005 and 2010 in the United States, 2.5% had computed tomography pulmonary angiogram (CTPA) done for the suspected pulmonary embolism (PE). Of these, about 3% had a confirmed PE by imaging studies.^1^, ^2^, ^3^, ^4^ Pulmonary embolism is the third cause of cardiovascular death, necessitating prompt, and accurate diagnosis.^1^, ^2^, ^3^, ^4^ Pulmonary embolism encompasses a wide range of clinical presentations, from asymptomatic to hemodynamic instability and sudden death. Symptoms of chest pain and shortness of breath, with or without hemodynamic compromise, may represent massive or submassive PE requiring immediate anticoagulation initiation.^5^, ^6^, ^7^ More importantly, massive PE needs to be treated promptly with fibrinolytic therapy. For selected patients with submassive PE, fibrinolysis may also be beneficial.^5^, ^6^, ^7^

Challengingly, the presentation of PE is similar to an acute coronary event and other cardiovascular emergencies such as aortic rupture, dissection, intramural hematoma, or penetrating aortic ulcer, as well as pneumothorax or esophageal perforation for which the use of thrombolytics and/or anticoagulation therapy may be detrimental. Measurement of fibrin D-dimer is routinely used to exclude the diagnosis of acute venous thromboembolism, but it cannot discriminate acute PE from aortic vascular emergencies or gastrointestinal perforation, which are also associated with elevated D-dimer.^8^^,^^9^ To differentiate between those medical entities CTPA is used, but it may not be readily available, is expensive, is unsafe for patients with renal disease, and contributes to radiation exposure.^9^ Indiscriminate use of CTPA has important implications for resource utilization.^4^ In addition, advances in CTPA technique have resulted in overdiagnosis of potentially less important PE, such as subsegmental, which may be anticoagulated without benefit and even with adverse events.^10^^,^^11^

Various combinations of electrocardiogram (ECG) abnormalities have been evaluated and found to be of some use for identifying PE, pulmonary hypertension, or PE-related mortality.^12^, ^13^, ^14^, ^15^, ^16^, ^17^, ^18^ A recently constructed ECG-based PE prediction model^19^ exhibited performance superior to Wells et al^20^ and Geneva^21^ criteria by the area under the receiver operating characteristic curve (AUROC) value, sensitivity, negative predictive value (NPV), and test accuracy, whereas the Geneva score presented superior specificity and positive predictive value (PPV).

Studies suggest that deep learning models can improve the detection of subtle, unnoticeable to a clinician, signals of ECG and in this way more effectively diagnose different cardiac conditions.^22^, ^23^, ^24^, ^25^, ^26^, ^27^, ^28^ We developed an artificial intelligence deep neural network (AI-DNN) for a 12-lead ECG analysis to recognize acute PE and severe categories of PE such as saddle pulmonary embolism (SADPE) or right ventricular strain pulmonary embolism (RVSPE).

## Patients and Methods

### Study Population

Patients across the Mayo Clinic Enterprise who had CTPA imaging with opacification of the entire pulmonary artery circulation between January 1, 1999, and December 31, 2020, were identified by searching a unified data platform. The Mayo Clinic Enterprise consists of numerous outpatient and inpatient locations using a common medical record system, including 3 tertiary referral centers (Rochester, Minnesota; Phoenix, Arizona; and Jacksonville, Florida) and regional Mayo Clinic Health System sites in Minnesota, Wisconsin, and Iowa. Institutional review board approval was obtained for this study.

All available ECGs were extracted, and the date of the CTPA study was used as the index date to assess the timing of the ECG. Electrocardiogram CTPA pairs were built considering the closest ECG to the CTPA date within ±6 hours, and if a patient had more than 1 CTPA, the initial study was kept. All ECGs were acquired as digital standard 12-lead, 10-second ECGs using a Marquette ECG machine (GE Healthcare).

### Analysis of PE Outcomes on CTPA Chest Reports

Natural language processing (NLP) algorithms were used to identify acute or new (acute on chronic) PE from CTPA chest imaging reports. Novel NLP algorithms were created and tested using the “simple NLP” program (http://iturrate.com/simpleNLP/). Random samples of radiology imaging reports were manually reviewed and interpreted as positive or negative results for acute PE by trained vascular providers (D.E.H., R.A.M., and W.E.W.). A review of the original CTPA images was not performed, but only verification that acute pulmonary emboli was described in the report.

Initial derivation and testing were performed on reiterative random samples of 100 CTPA chest reports from the overall database with modification and retesting until maximum accuracy was obtained. To increase the manual review of CTPA reports with positive findings, the algorithm was then applied to the entire database, and results from 100 random reports (50 positive and 50 negative results by the NLP) were manually reviewed with modifications to the algorithm, and retesting on series of 100 random reports was performed until we reached maximum accuracy. For the final validation, the NLP algorithm was run on the entire database, and a random sample of 500 positive and 500 negative (by NLP) reports were manually and independently reviewed to confirm the accuracy of the algorithm. In total, 2000 CTPA reports were manually reviewed through the process of derivation and validation. Within this entire cohort, the sensitivity of the NLP was 99.3% and the specificity 99.1% for findings of new acute pulmonary emboli. A similar process of derivation and validation was performed to create algorithms that identified “any” pulmonary emboli, regardless of age or chronicity. The sensitivity of this algorithm was 100% with a specificity of 98%. Natural language processing algorithms were also derived and tested to identify patients with SADPE or RVSPE. Validation of these algorithms was performed in a random sample of 100 CTPA reports. The algorithm for SADPE was 100% sensitive and specific and the algorithm for RVSPE was 71% sensitive and 100% specific.

### Model Development

Computed tomography pulmonary angiogram reports negative for PE and positive for acute PE were included to generate the final annotations for our cohorts. Subacute, chronic, and PE of undetermined age were excluded from analyses. Patients were allocated to the training, validation, and testing sets in a 7:1:2 ratio via outcome-stratified (eg, positive/negative PE) random sampling; each patient was uniquely assigned to a single set.

Although the initial convolutional neural network architecture was similar to the already published model by our group for predicting the patient’s sex^29^ and diagnosing cardiac amyloidosis,^24^ a transfer learning technique, where weights of the preexisting network (eg, predicting patient’s sex model) were either updated very slowly or frozen entirely, was used improving our results. Each ECG was considered a matrix of the following dimensions: 12×5000 (representing 12 leads for a 10-second duration sampled at 500 Hz), ECGs that were initially sampled at 250Hz were upsampled to 500 Hz using the “Resample” function of the SciPy python package^30^ where the first dimension being spatial and representing the different ECG leads, and the second dimension being temporal.

Hyperparameters such as learning rate (1×^10−3^), batch size (64), and number of layers to be frozen (5) were tuned based on the validation set performance. The AUROC was calculated after each epoch for both training and validation sets. After training completion, the model with the highest AUROC in the validation set was evaluated on the testing set. An early stopping strategy controlled the number of training epochs, where the network weights were updated as long as the loss improved. After 10 epochs without improvement, training was halted.

Electrocardiograms were classified as positive for PE when their probability score was greater than or equal to the threshold (eg, Youden index^31^ chosen from the receiver operating characteristics curve of the validation set). This threshold can be tuned based on different clinical scenarios, for instance, prioritizing sensitivity over specificity. In this study, we sought a balance between the 2 and selected the Youden index as the optimal operating threshold.

### AI-DNN of ECG to Detect PE

Model efficacy was assessed by detecting PE using the closest 12-lead ECG within ±6 hours. All performance metrics were based on the testing set; standard measures of diagnostic performance (AUROC, sensitivity, and specificity) were computed as well as their 95% CIs. To determine the area under the curve, the large sample approximation of the DeLong method optimized by Sun and Xu was used.^32^ The diagnostic odds ratio, which is the ratio of the positive likelihood ratio (sensitivity/[1−specificity]) to the negative likelihood ratio ([1−sensitivity]/specificity), and its associated 95% CI was also calculated. The performance of the models was assessed in the entire CTPA report cohort and the subsets of patients according to age, sex, and combination of them. We also performed a subanalysis based on PE categories: acute PE, RVSPE, and SADPE.

## Results

### Study Population

A total of 257,969 CTPA radiology reports with NLP outcomes were identified in 181,098 patients. Among these, we identified 199,545 with ECGs. After setting our model to eliminate radiology reports who had ECG performed more than 6 hours before or after CTPA, had multiple radiology CTPA reports, had no research authorization, patients younger than 18 years, those with missing date of birth or problematic age assessment, corrupted ECG, or had unclear CT description, and PE of not-acute or undetermined age emboli, we ended up with 79,894 CTPA-ECG pairs (Supplemental Figure 1A, available online at https://www.mcpdigitalhealth.org/). Analyzed CTPA reports included the following 6 types of radiology images: CT chest angiogram and pulmonary arteries with intravenous (IV) contrast, CT chest angiogram with IV contrast, CT chest pulmonary embolus angiogram, CT chest abdomen pelvis angiogram with IV contrast, CT chest abdomen angiogram with IV contrast, and CT cardiac angiogram triple rule out with contrast. The number of patients, sex, and age distribution for every CTPA radiology study type and the whole cohort are summarized in Supplemental Figures 1B, C, and 2 (available online at https://www.mcpdigitalhealth.org/).

The final cohort consisted of 79,894 patients: 53,692 (67.2%) were evaluated at the emergency department, 17,624 (22.1%) as an inpatient, and 7892 (9.9%) as an outpatient; for the remaining 686 (0.9%), the clinical setting cannot be determined. There were 7423 (9.29%) with acute PE in our cohort. Patients with CTPA reports positive for acute PE were older with a lower proportion of female (mean age, 63.7±15.9 years, 46.1% female) compared with 72,471 patients who had negative results (mean age, 60.5±17.6 years, 52.3% female) (Table 1). Within the group of patients with acute PE, 4,242 (57.1%) had D-dimer level measured, revealing 4101 (96.7% tested) with positive result. Of 72,471 patients with negative CTPA, 30,555 (42.2%) had testing for D-dimer, which found positive results in 25,979 (85.0% of tested). D-dimer testing was considered to be positive based on the specific threshold used for a specific assay because multiple assay types were used over the study timeframe. Supplemental Figure 3A-D (available online at https://www.mcpdigitalhealth.org/) shows sex and age distribution according to the type of CTPA radiology study for patients with acute PE and patients with negative report for any PE, as well as time distribution between CTPA and ECG, respectively.

**Table 1:** Baseline Characteristics Across Analysis Cohort^a^^,^^b^

Demographic characteristic	PE positive	PE negative	Total
Any acute pulmonary embolism (PE)			
Total No. of patients	7423 (9.29)	72,471 (90.71)	79,894
Age (y), mean ± SD	63.75±15.93	60.47±17.60	
Sex, n (%)			
Female	3423 (8.29)	37,890 (91.71)	41,313
Male	4000 (10.37)	34,581 (89.63)	38,581
D-dimer measured, n (% of total)	4242 (57.1)	30,555 (42.2)	34,797
D-dimer positive, n (% of measured)	4101 (96.7)	25,979 (85.0)	30,080
D-dimer negative, n (% of measured)	141 (3.3)	4576 (15.0)	4717
Saddle PE or PE with the right ventricular strain			
Total No. of patients	1138	72,471	73,609
Age (y), mean ± SD	65.56±14.06	60.47±17.60	
Sex, n (%)			
Female	520 (1.48)	34,581 (98.52)	35,101
Male	618 (1.60)	37,890 (98.40)	38,508

a Abbreviation: SD, standard deviation.

b Values n, (%). Mean ± SD.

High-risk PE categories such as RVSPE or SADPE were identified in 1138 (1.6%) patients (mean age, 65.6±14.1 years, 45.7% of females) (Table 1). The sex and age distribution of patients with RVSPE or SADPE in relation to the patients with CTPA report negative for PE are shown in Supplemental Figure 4A, B (available online at https://www.mcpdigitalhealth.org/), respectively.

### Model Evaluation

Among 79,894 CTPA-ECG pairs consisting of patients with acute PE (of any severity, n=7423), those with negative results for PE (72,471) was used to evaluate prediction of acute PE. The number of patients with negative and positive study results for PE used for training, validation, and testing data sets is summarized in Table 2. The AI-DNN prediction of acute PE was calculated as AUROC of 0.69 (95% CI, 0.68-0.71) (Figure). The sensitivity was 63.5% and the specificity was 64.7%, with a PPV of 15.6% and NPV of 94.5%. The performance of the algorithm to detect acute PE tabulated across a range of age (younger or older than 50 years) and sex is summarized in Table 3. The lowest sensitivity of our algorithm was within the group of women younger than 50 years (possibly because of relatively low number of women of this age with positive CTPA for acute PE).

**Table 2:** Distribution of Positive and Negative testing for PE in Training Validation and Testing Sets

Cohort, n (%)	Training			Validation			Testing		
	Positive	Negative	Total	Positive	Negative	Total	Positive	Negative	Total (%)
Acute PE	5196 (9.29)	50,729 (90.71)	55,925 (100)	735 (9.29)	7174 (90.71)	7909 (100)	1492 (9.29)	14,568 (90.71)	16,060 (100)
SADPE or RVSPE	797 (1.55)	50,729 (98.45)	51,526 (100)	113 (1.55)	7174 (98.45)	7287 (100)	228 (1.54)	14,568 (98.46	14,796 (100)

Abbreviations: PE, pulmonary embolism; RVSPE, right ventricular strain pulmonary embolism; SADPE, saddle pulmonary embolism.

**Figure:**
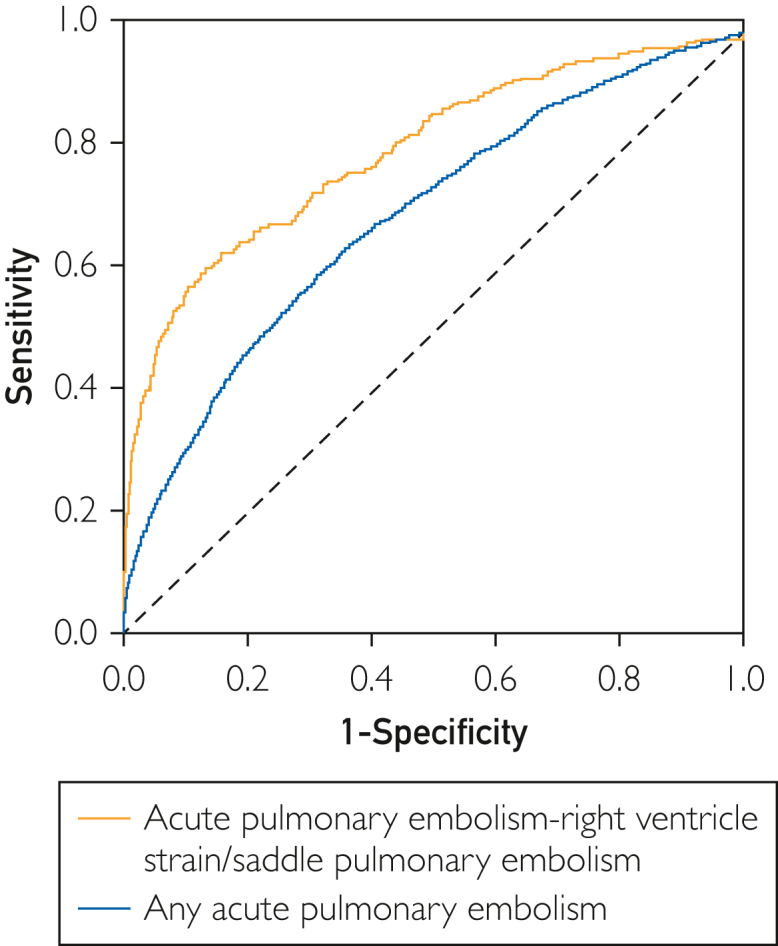
Receiver operating characteristics curve and resulting area under the curve for patients with any acute pulmonary embolism (red line) and subgroup of patients with severe pulmonary embolism with right ventricle strain and/or with saddle pulmonary embolism (blue line).

**Table 3:** Performance of the Acute Pulmonary Embolism Detection Algorithm Across Age and Sex Subgroups

Group	N	AUROC	Sensitivity (95% CI) (%)	Specificity (95% CI) (%)		OR (95% CI)
Age <50 y	4159	0.672 (0.638-0.705)	55.4 (49.1-61.5)	69.2 (67.8-70.7)		2.8 (2.2-3.6)
Age ≥50 y	11,901	0.693 (0.677-0.709)	65.3 (62.5-67.9)	63.0 (62.1-63.9)		3.2 (2.8-3.6)
Female	8296	0.684 (0.663-0.705)	55.3 (51.5-59.1)	70.3 (69.2-71.3)		2.9 (2.5-3.4)
Female <50 y	2409	0.652 (0.607-0.697)	41.1 (32.5-50.1)	74.7 (72.9-76.5)		2.1 (1.4-3.0)
Female ≥50 y	5887	0.687 (0.663-0.711)	58.7 (54.4-62.9)	68.4 (67.1-69.6)		3.1 (2.6-3.7)
Male	7764	0.696 (0.676-0.716)	70.2 (66.9-73.3)	58.5 (57.3-59.6)		3.3 (2.8-3.9)
Male <50 y	1750	0.692 (0.642-0.742)	69.5 (60.8-77.2)	61.5 (59.1-63.9)		3.6 (2.5-5.3)
Male ≥50 y	6014	0.695 (0.673-0.716)	70.3 (66.8-73.7)	57.6 (56.2-58.9)		3.2 (2.7-3.8)
Overall	16,060	0.693 (0.673-0.716)	63.5 (61.0-66.0)	64.7 (63.9-65.4)		3.2 (2.9-3.6)
					OR	

Abbreviations: AUROC, area under the receiver operating characteristic curve; OR, odds ratio.

To assess whether advancing CT technique has an impact on our model performance, the prediction of acute PE was calculated for a subgroup of patients with any acute PE diagnosed with the past 10 years (AUROC=0.69) (Supplemental Figure 5A, available online at https://www.mcpdigitalhealth.org/) and for the past 5 years (AUROC=0.68) (Supplemental Figure 5B).

### Acute PE with Saddle or Right Ventricular Strain

To assess the prediction of severe PE category such as SADPE or RVSPE, a cohort of 73,609 consisting of either (n=1138), and patients with negative-result CTPA for PE (n=72,471) was used. The number of cases with negative and positive study results for PE used for training, validation, and testing data sets for the algorithm specific for this cohort are summarized in Table 2. The AI-DNN prediction of RVSPE or SADPE was assessed as AUROC of 0.84 (95% CI, 0.81-0.86) (Figure), with a sensitivity of 80.8%, specificity of 64.7.8%, PPV of 3.5%, and NPV of 99.5%. The performance of the prediction for the presence of RVSPE or SADPE using AI-DNN developed from the RVSPE or SADPE cohort was similar (Supplemental Figure 6, available online at https://www.mcpdigitalhealth.org/). To assess the effect of advancing CT technique on the performance of our model, the prediction for the presence of RVSPE or SADPE was calculated for a subgroup of patients diagnosed within the past 10 and within 5 years and found them very similar to the whole cohort (Supplemental Figure 7A, B, respectively, available online at https://www.mcpdigitalhealth.org/).

## Discussion

The goal of this study was to optimally train an AI-DNN model solely using ECG waveforms, in a gold standard cohort of patients with definitive presence or absence of PE, which could be applied in future diagnostic accuracy studies of PE. In the whole cohort that included all types and severities (including subsegmental PE), the prediction of PE was modest with the AUROC approaching 0.7. In cases of severe PE with high thrombus burden or with right ventricle dysfunction/injury, the AI-DNN prediction improved to AUROC value of 0.84. The performance of our AI model was persistent during the study period because the subanalysis of PE prediction for any acute PE or high-risk PE (RVSPE or SADPE) diagnosed within the past 10 and 5 years found very similar results to those of the whole cohort diagnosed between 1999 and 2020. We also found similar prediction for high-risk PE (RVSPE or SADPE) when we used AI-DNN developed specifically from the cohort of patients with SADPE or RVSPE; slightly lower AUROC value when using this second model (0.80 vs 0.84) is most likely related to the smaller number of patients (for both negative control and positive PE groups) and therefore reduced chance to capture ECG features related to PE.

Our findings add to previous studies that use ECG abnormalities detected by clinicians to predict PE.^16^, ^17^, ^18^ Daniel et al^16^ developed an ECG scoring system and found that only patients with severe PE had a significantly higher ECG score. Incorporating additional ECG abnormalities by Su et al^19^ further improved PE prediction. The prediction of acute PE in this study based on raw ECG waveform analysis resulted in a higher discriminatory index (despite the inclusion of all types of PE) than the study published by Somani et al^27^ (AUROC 0.59). However, a recent relatively small study of AI model for predicting PE using a 12-lead ECG reported higher AUROC of 0.75 and a specificity of 100%.^28^ We compared the performance of our current model to predict any acute PE, or PE with right ventricle strain (RVSPE) or saddle PE (SADPE), to the results of prior studies applying machine learning models^27^^,^^28^ analyzing an ECG waveform only or in combination with clinical data (fusion model), and to the results of electrocardiography models for PE diagnosis^19^ (see Supplemental Table, available online at https://www.mcpdigitalhealth.org/).

A key finding of this study is the high NPVs for excluding either all acute PE (NPV=94.54%) or specifically severe PE (NPV=99.52%). In the setting of chest pain, dyspnea, or hemodynamic instability, the prompt and accurate diagnosis of PE is central to the delivery of appropriate therapy. This is particularly important for high-risk PE when immediate implementation of anticoagulation before PE is confirmed by CTPA is recommended. In such clinical settings, the differential diagnosis includes aortic rupture, dissection, aortic intramural hematoma, or penetrating ulcer, for which anticoagulation therapy is not helpful but could be extremally dangerous, and D-dimer measurement cannot discriminate acute PE from aortic vascular emergencies^8^^,^^9^; however, using our model allows to instantly eliminate the necessity for immediate anticoagulation implementation. On the contrary, accurate exclusion of PE by our AI-DNN ECG analysis might reduce the necessity for D-dimer testing when PE is in the differential diagnosis. In general, tools helpful in distinguishing these diseases by accurate bedside assessment would be immensely useful, particularly in the emergency department setting. Moreover, ECG requisition is nearly universal in these settings. Extending the ECG interpretation by adding an AI-based analysis algorithm for PE probability would be easily implemented, timesaving, and a resource-conserving addition to the bedside clinical assessment.

As might be suspected, the results of our study support the notion that ECG changes are proportional to the severity of PE reflecting the hemodynamic consequences of thromboembolic PE burden. Electrocardiogram signals likely provide information reflecting both cardiomyocyte and pulmonary arterial circulatory physiology. This principle represents the analogy to ECG’s usefulness in coronary artery disease. Although the ECG is not likely able to directly identify pulmonary arterial thrombotic disease, the impact of this obstruction on cardiac myocyte function might be anticipated. Our observation that the AI-DNN model demonstrates increasing performance with increasing PE embolic burden implies that it accurately identifies ECG waveform changes related to PE and generates information beyond what is achieved by traditional ECG analysis. However, we suspect a limitation to this diagnostic approach will be the identification of smaller or perhaps nonocclusive PE that may not alter cardiac physiology.

Previous studies reported that adding demographic and clinical data traditionally associated with an increased likelihood of PE significantly improves the prediction of PE.^27^ Our model performance may likewise be enhanced by adding demographic and clinical parameters, which will be the goal of future studies where a more real-world cohort can be examined (all patients with suspected PE rather than only high-risk patients who ultimately got CTPA). Despite the limited demographic data analyzed in this study, these preliminary results are encouraging both from a negative and positive predictive value standpoint. Depending on the future studies and the ultimate diagnostic use of AI ECG algorithms, the sensitivity or specificity could be prioritized by altering model parameters. Patients with positive findings in our study were older compared with those with negative testing, a well-known demographic characteristic of patients with PE.^1^, ^2^, ^3^, ^4^

The strength of this study is that it used a large number of CTPA studies that visualized the entire system of pulmonary artery circulation and whose reports were scrutinized by newly developed, highly accurate NLP models. The NPV achieved by our AI-DNN analysis of ECG to exclude severe PE is similar to the NPV of D-dimer testing. This is achieved with the use of the bedside method that is very often used, easy, quick, noninvasive, and inexpensive. In contrast to typical clinical risk stratification tools that are not typically applied to (or validated in) inpatients, this AI ECG algorithm may be useful for both inpatients and outpatients. Another advantage of an AI ECG approach for risk stratification of PE is that it uses an already integrated clinical tool in the workup of cardiopulmonary symptoms and can be uniformly and objectively applied, which is a significant limitation to current risk stratification algorithms.^33^^,^^34^

Our study also has some limitations that need to be considered. First, the accuracy of the diagnosis of PE was not verified by secondary radiology evaluation. Second, we had subsegmental PE cases in our CTPA cohort, which were unlikely to be associated with detectable ECG abnormalities and might not even require anticoagulation. Third, very high NPV for high-risk PE (RVSPE or SADPE) is partly driven by the low prevalence of these PE types among the overall cohort. Fourth, lack the information on the final diagnosis in patients without PE may decrease reliability of the control group. Finally, the performance of our algorithm was derived from a group of patients with a relatively high-risk for PE, and therefore, true diagnostic accuracy testing needs to be performed in a wider group of patients with “suspected PE” seen at the emergency department who undergo ECG testing to determine the model use in such setting, and this is currently our ongoing project.

## Conclusion

In conclusion, an AI-based analysis of 12-lead ECG in a large cohort of patients with CTPA, without any additional discriminating demographic or clinical variables, reported a modest ability to detect acute PE in a cohort of patients who underwent CTPA, with improved accuracy for high-risk PE. Moreover, AI-DNN ECG waveform analysis provides a fast, objective, and consistent way to risk stratify patients for PE.

## Potential Competing Interests

Given his role as Editor-in-Chief, Dr Francisco Lopez-Jimenez had no involvement in the peer-review of this article and had no access to information regarding its peer-review. Full responsibility for the editorial process for this article was delegated to an unaffiliated Editor. Dr Harmon reports grants from NIH StARR R38 grant (NHLBI): NIH 5R38HL150086-02. Dr Houghton reports grants from Gordan & Betty Moore Foundation, American Society of Hematology, Bayer, Hemostasis & Thrombosis Research Society, Noaber Foundation, Veralox to the institution. The other authors report no competing interests.
