# Supplementary material for: Electrocardiogram Signal Analysis With a Machine Learning Model Predicts the Presence of Pulmonary Embolism With Accuracy Dependent on Embolism Burden
Source: Mayo Clin Proc Digit Health. 2024 May 24;2(3):453–62. doi: 10.1016/j.mcpdig.2024.03.009 (PMC11975982; doi:10.1016/j.mcpdig.2024.03.009)
Supplement: Supplemental Figure 1-7 and Supplemental Table [file mmc1.pdf]

Figure S1A. Mayo Clinic network cohort retrospectively identified by natural language processing (NLP)

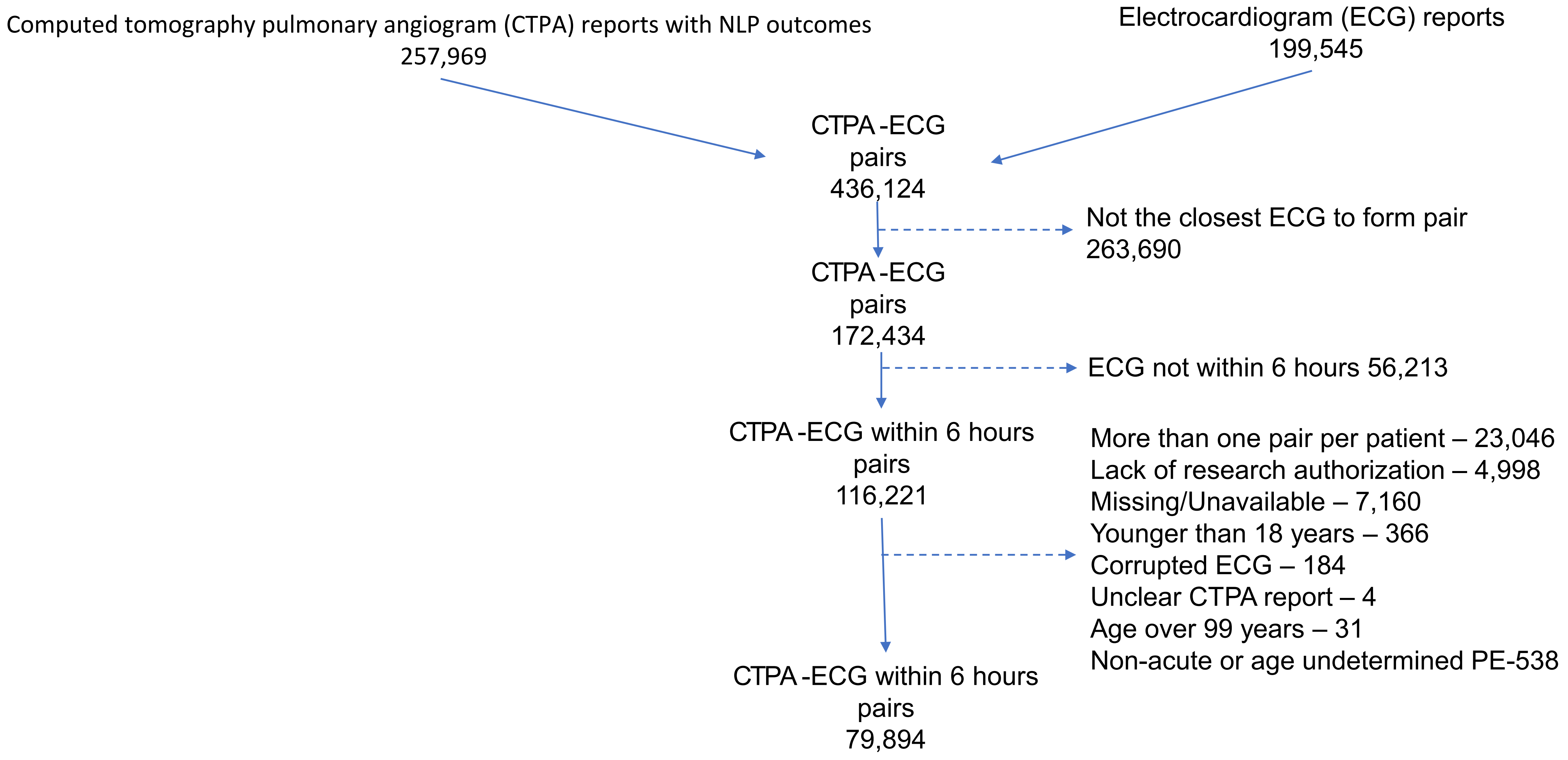

Figure S1B. Number of reports for each computed tomography pulmonary angiogram type (panel A), and with sex profile (panel B).

Test Description	Number of patients
T CHEST ANGIOGRAM AND PULMONARY ARTERIES WITH IV CONTRAST	41,549
CT CHEST ANGIOGRAM WITH IV CONTRAST	19,211
CT CHEST PULMONARY EMBOLISM ANGIOGRAM	9,915
CT CHEST ABDOMEN PELVIS ANGIOGRAM WITH IV CONTRAST	4,690
CT CHEST ABDOMEN ANGIOGRAM WITH IV CONTRAST	3,784
CT CARDIAC ANGIOGRAM TRIPLE RULE OUT WITH IV CONTRAST	1,283
TOTAL	80,432

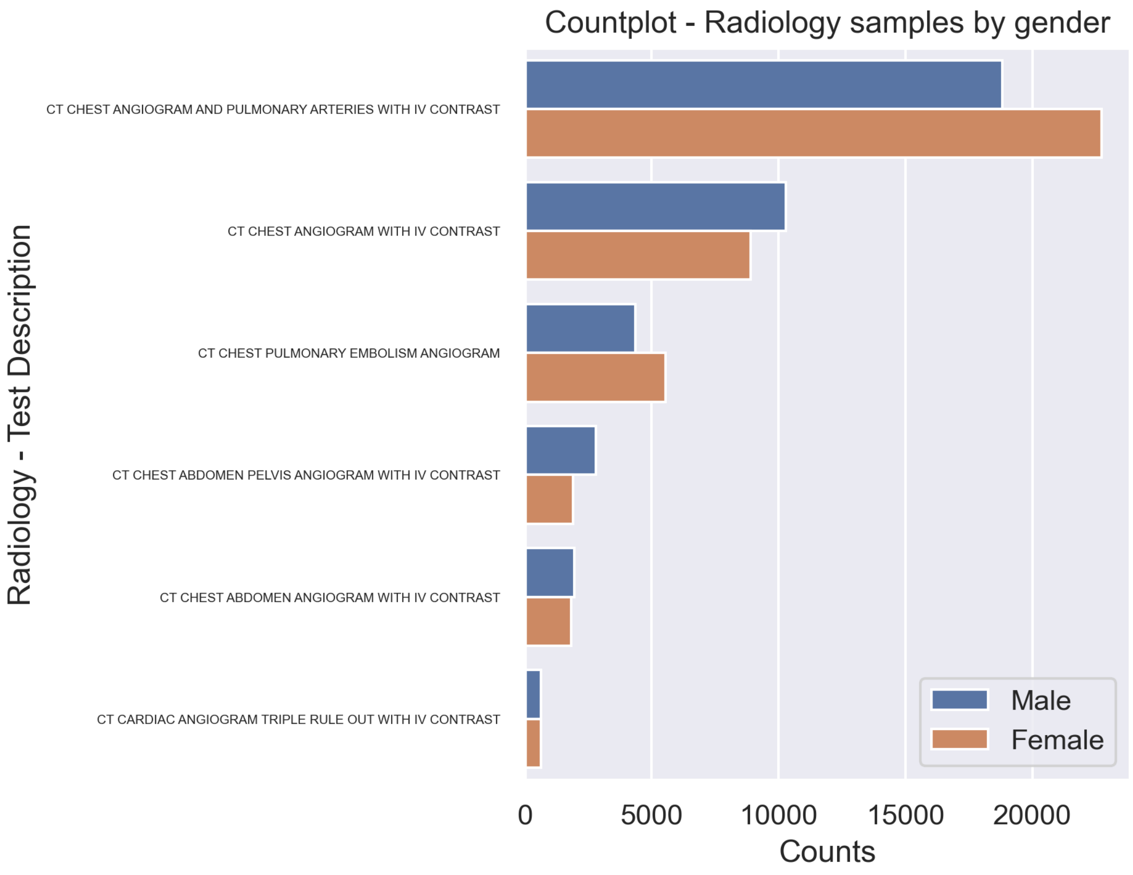

Figure S1C. Age of patients for every type of computed tomography pulmonary angiogram

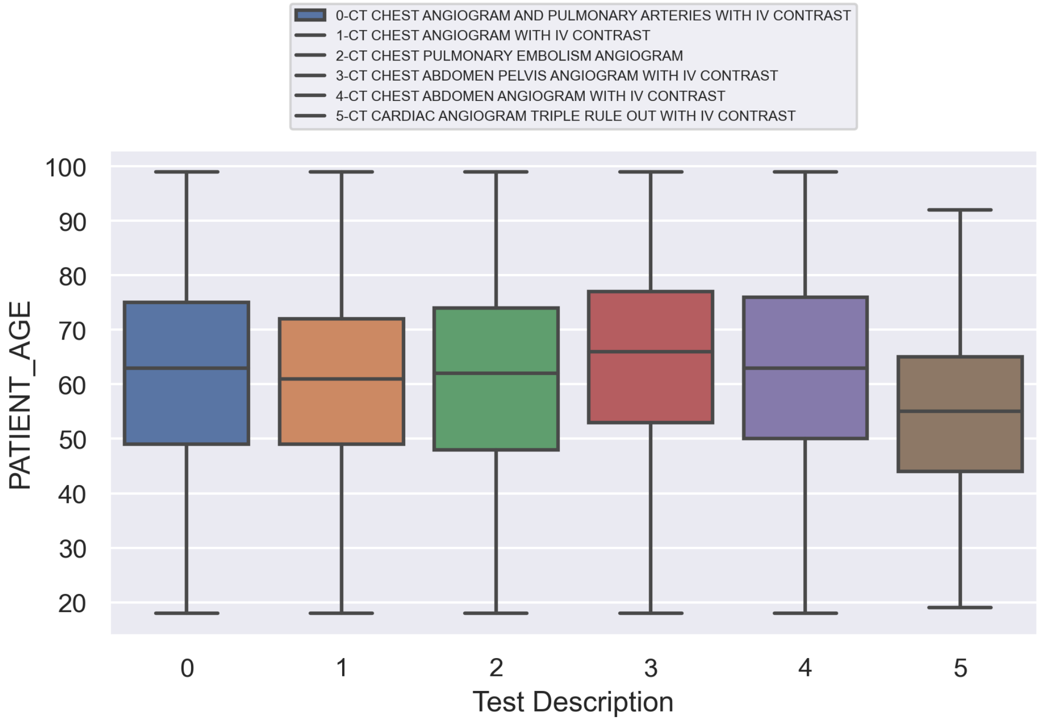

	Age (years)			
Test Description	Mean	Standard Deviation	Minimal	Maximal
CT CHEST ANGIOGRAM AND PULMONARY ARTERIES WITH IV CONTRAST	60.92	17.86	18	99
CT CHEST ANGIOGRAM WITH IV CONTRAST	60.05	16.75	18	99
CT CHEST PULMONARY EMBOLISM ANGIOGRAM	60.28	18.06	18	99
CT CHEST ABDOMEN PELVIS ANGIOGRAM WITH IV CONTRAST	64.32	16.22	18	99
CT CHEST ABDOMEN ANGIOGRAM WITH IV CONTRAST	62.04	16.55	18	99
CT CARDIAC ANGIOGRAM TRIPLE RULE OUT WITH IV CONTRAST	54.75	54.75	18	99
All Tests	60.79	17.48	18	99

Figure S2. Age and sex distribution of patients with computed tomography pulmonary angiogram reports.

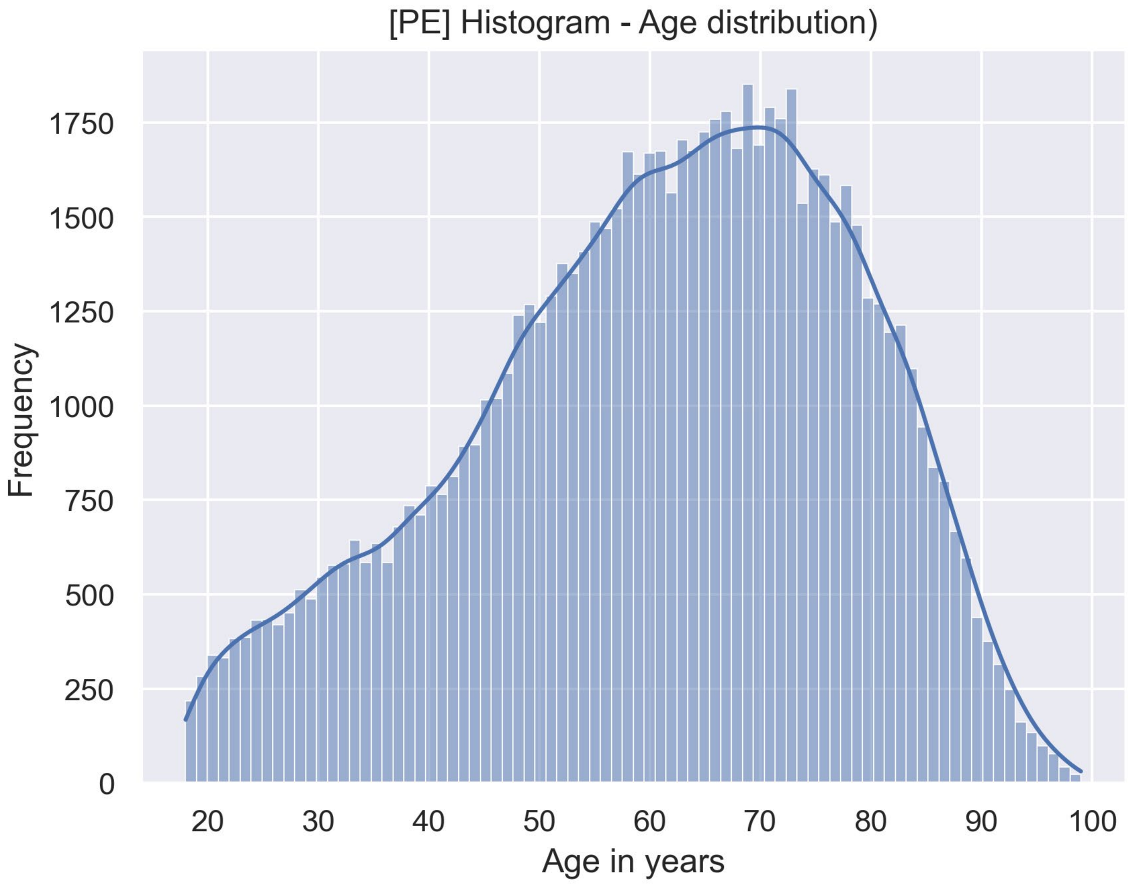

Age	Mean	std	Min	Max
Female	59.78	18.53	18	99
Male	61.86	16.21	18	99
Population	60.79	17.48	18	99

Sex	Count	[%]
Female	41,561	51.67%
Male	38,871	48.33%
TOTAL	80,432	100.00%

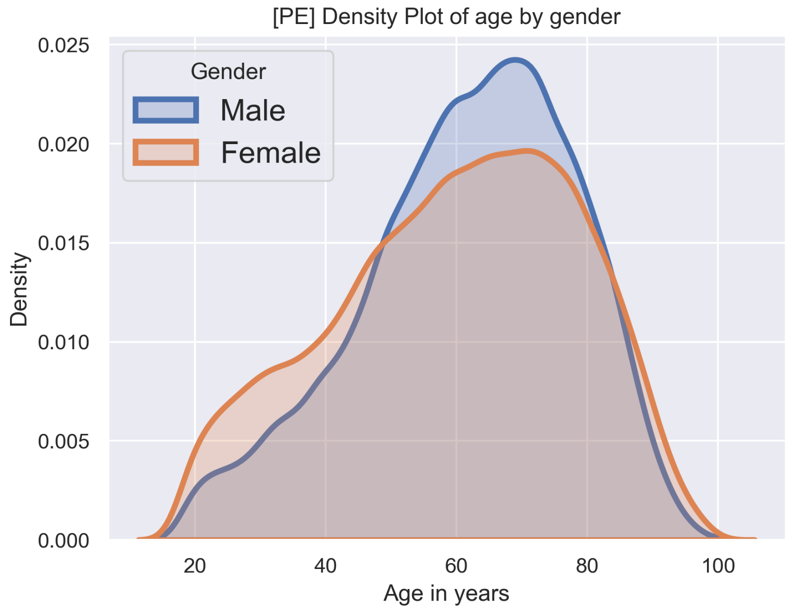

Figure S3A. Age and sex distribution of patients with positive reports for acute pulmonary embolism and negative for any pulmonary embolism according to the type of computed tomography pulmonary angiogram

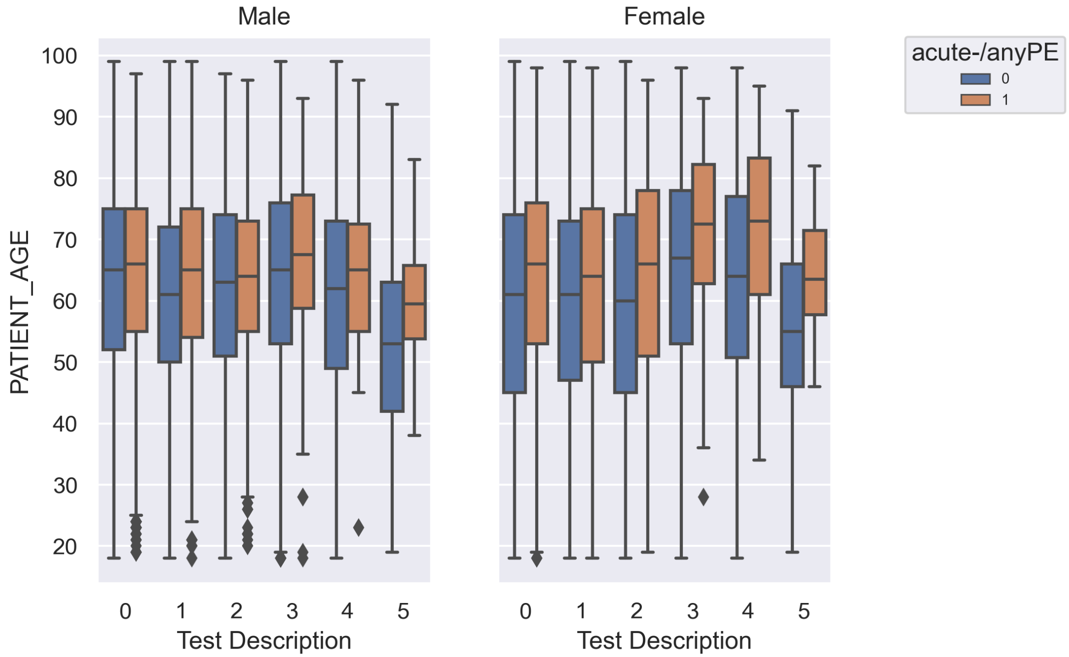

All Samples - Age	Male								Female							
	anyPE (-)				acutePe (+)				anyPe (-)				acutePe (+)			
Test Description	Mean	Std	Min	Max	Mean	Std	Min	Max	Mean	Std	Min	Max	Mean	Std	Min	Max
CT CHEST ANGIOGRAM AND PULMONARY ARTERIES WITH IV CONTRAST	62.47	16.79	18	99	64.27	14.39	19	97	58.95	18.92	18	99	63.78	17.04	18	98
CT CHEST ANGIOGRAM WITH IV CONTRAST	60.26	15.49	18	99	63.48	15.27	18	99	59.31	18.08	18	99	61.95	17.75	18	98
CT CHEST PULMONARY EMBOLISM ANGIOGRAM	61.50	16.67	18	97	62.86	14.33	20	96	58.75	19.25	18	99	63.18	18.26	19	96
CT CHEST ABDOMEN PELVIS ANGIOGRAM WITH IV CONTRAST	63.77	15.77	18	99	66.39	16.19	18	93	64.82	16.82	18	98	69.95	16.12	28	93
CT CHEST ABDOMEN ANGIOGRAM WITH IV CONTRAST	60.55	16.36	18	99	65.37	14.63	23	96	63.37	16.63	18	98	69.97	17.42	34	95
CT CARDIAC ANGIOGRAM TRIPLE RULE OUT WITH IV CONTRAST	53.53	15.12	19	92	59.95	11.01	38	83	55.63	15.03	19	91	64.92	10.59	46	82
TOTAL	61.60	16.37	18	99	63.95	14.58	18	99	59.44	18.60	18	99	63.51	17.37	18	98

Figure S3B. Comparison of age distribution and sex profile of patients with computed tomography pulmonary angiogram report positive for acute pulmonary embolism [acutrPE(+)] and report negative for any PE [anyPE(-)].

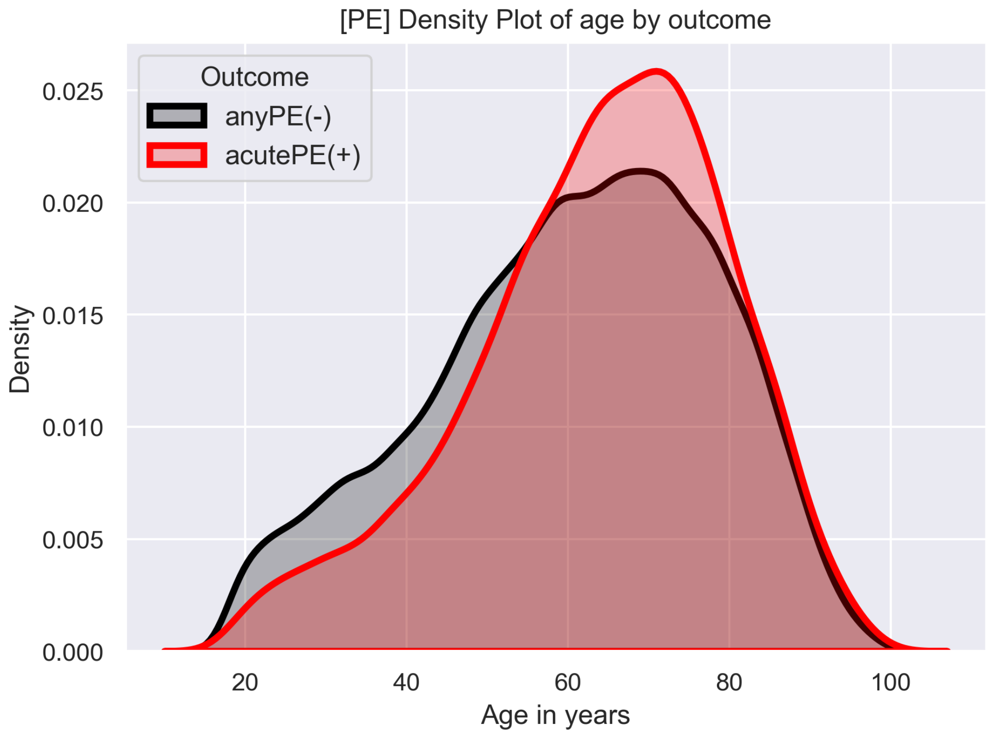

	Acute PE		Negative for any PE	
	mean	std	mean	std
Female	63.51	17.37	59.44	18.60
Male	63.95	14.58	61.60	16.37
Population	63.75	15.93	60.47	17.60

Figure S3C. Comparison of sex distribution of patients with computed tomography pulmonary angiogram report positive for acute pulmonary embolism (PE) and report negative for any PE.

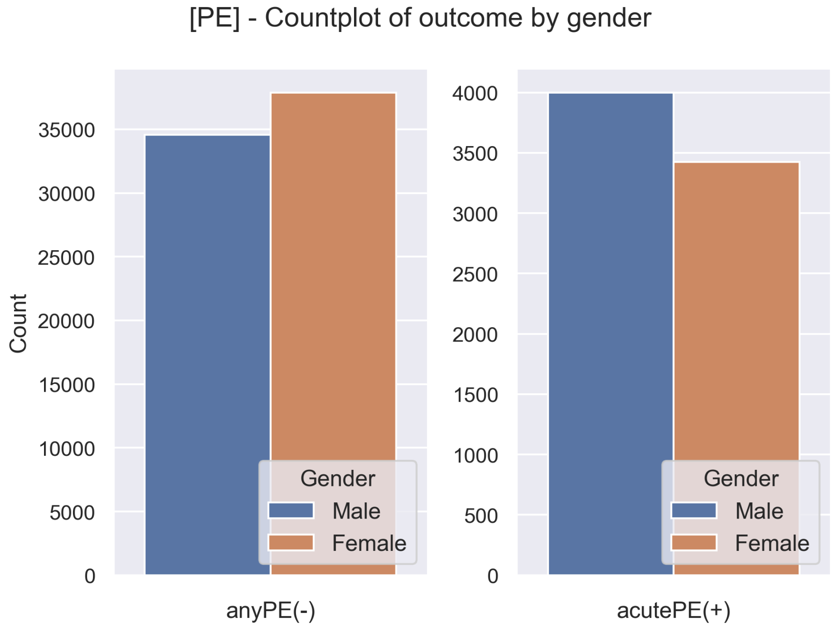

	Acute PE (%)	%	Negative for any PE (%)	%	Total
Female	3,423 (46.1%)	8.29	37,890 (52.3%)	91.71	41,313
Male	4,000 (53.9%)	10.37	34,581 (47.7%)	89.63	38,581
Total	7,423	9.29	72,471	90.71	79,894

Figure S3D. Distribution of timing between acute PE diagnosis and ECG testing in TCPA and ECG pairs of computed tomography pulmonary angiogram report cohort

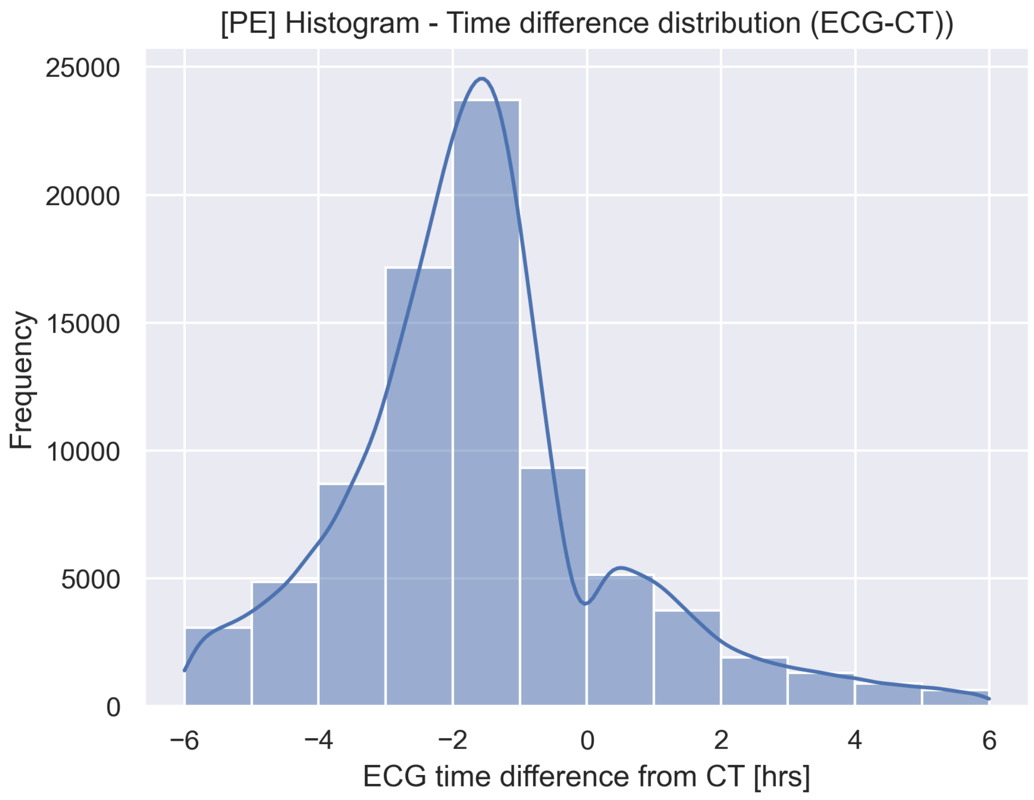

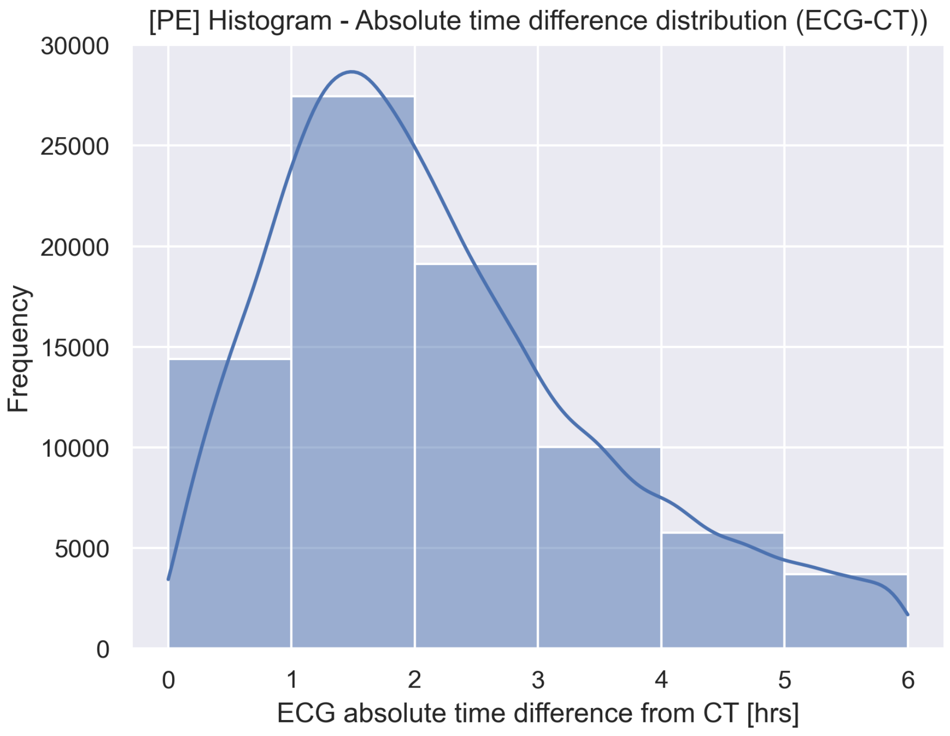

Figure S4A. Age distribution in patients with saddle PE (SADPE) or PE with the right ventricular strain (RVSPE) compared to the patients with report negative for any PE.

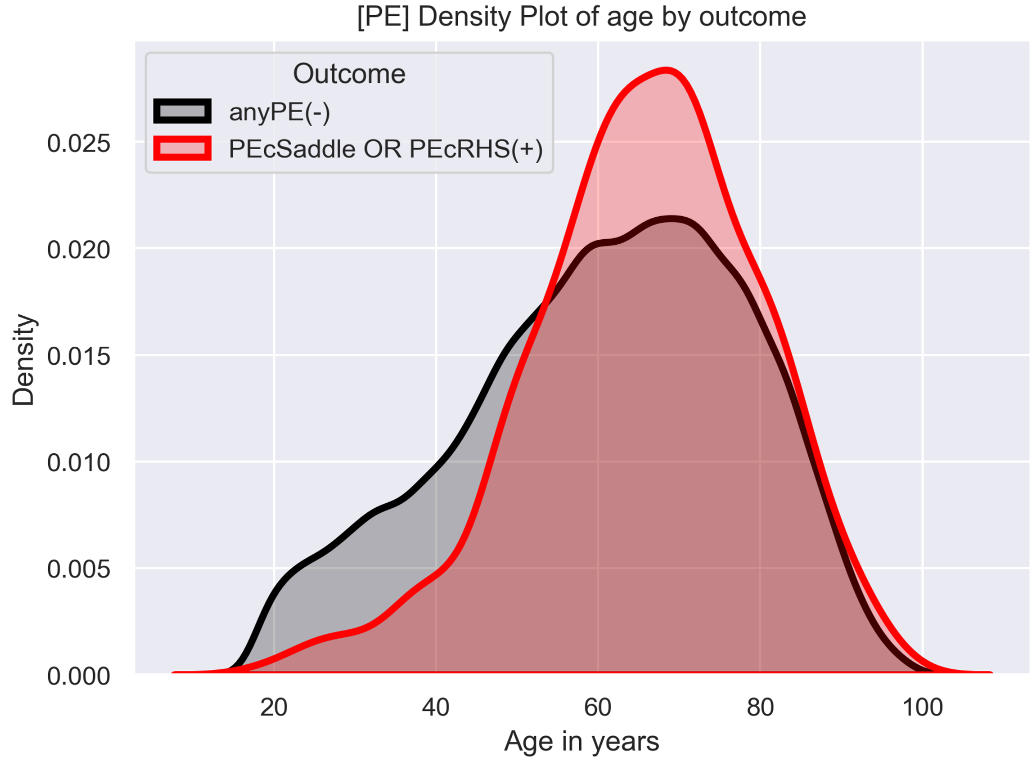

	SADPE or RVSPE		Negative for any PE	
	mean	std	mean	std
Female	66.29	15.25	59.44	18.60
Male	64.95	12.94	61.60	16.37
Population	65.56	14.06	60.47	17.60

Figure S4B. Sex distribution in patients with saddle pulmonary embolism (SAPE), pulmonary embolism with right ventricular strain (RVSPE) compared to patients with negative computed tomography pulmonary angiogram report for pulmonary embolism (no PE)

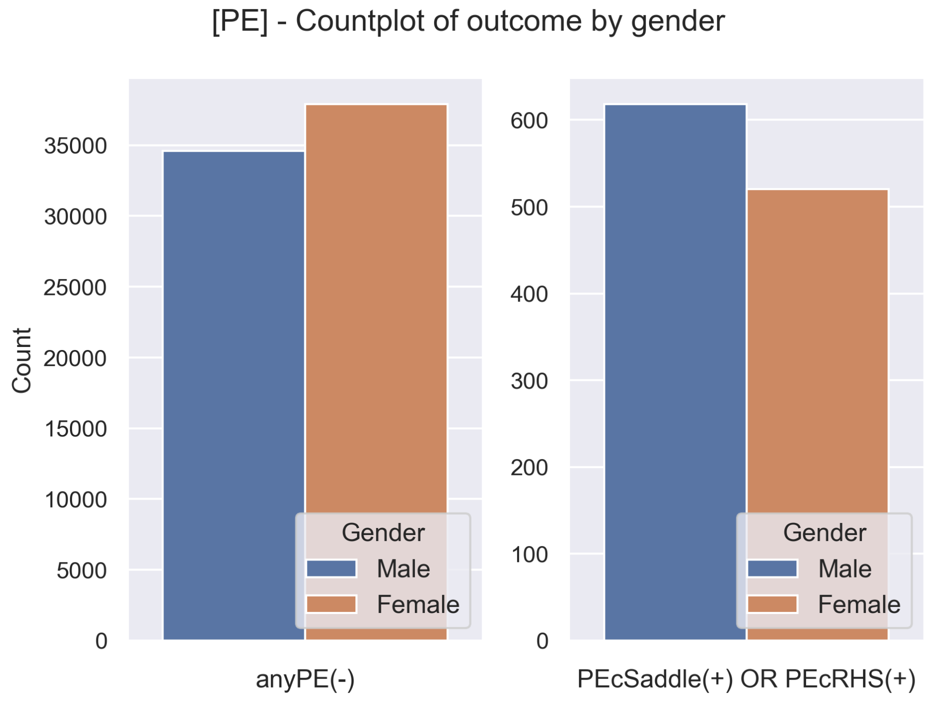

	SAPE or RVSPE (%)	[%]	No PE (%)	[%]	Total
Female	520 (45.7)	1.35%	37,890 (52.3)	98.65%	38,410
Male	618 (54.3)	1.76%	34,581 (47.3)	98.24%	35,199
Total	1,138	1.55%	72,471	98.45%	73,609

Figure S5. Receiver Operating Characteristics Curve and Resulting Area Under the Curve for patients with Any Acute Pulmonary Embolism diagnosed between 2010 and 2020 (last 10 years; panel A) and between 2015 and 2020 (last 5 years; panel B).

A

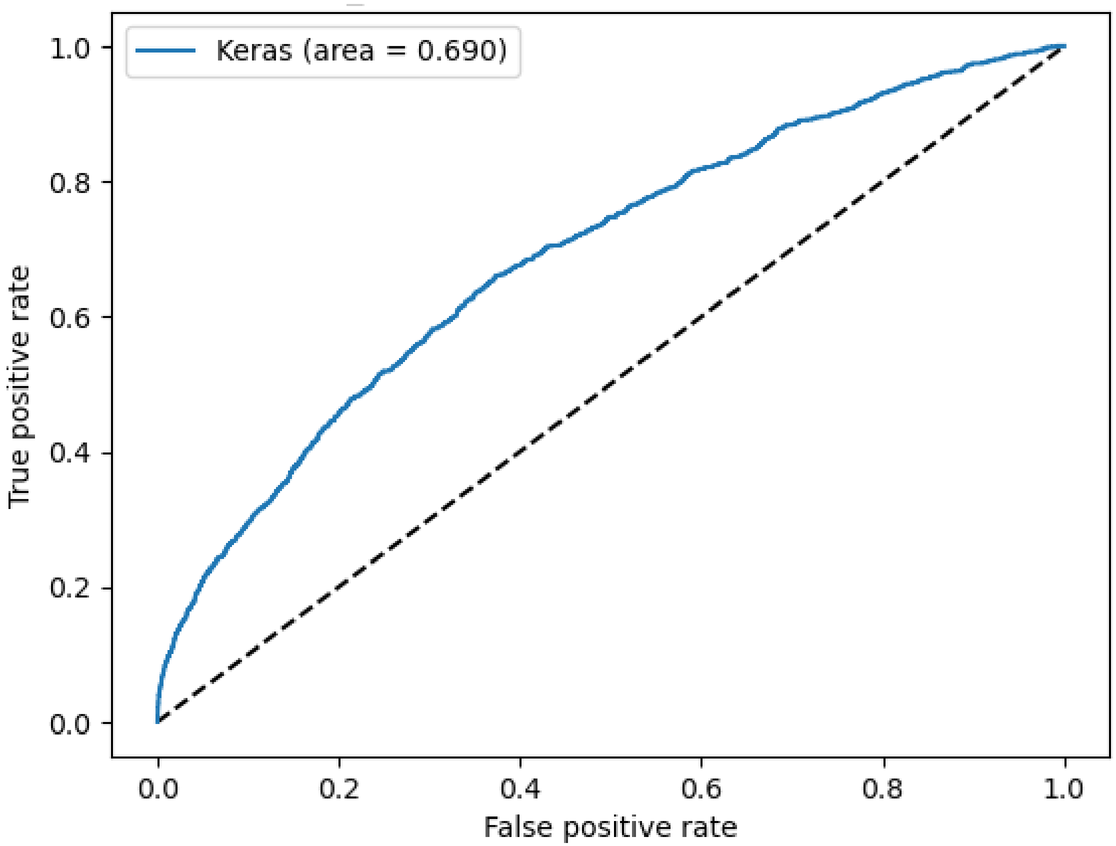

AUROC of 0.69, 95% CI 0.67-0.71 . The sensitivity was 66.1% and the specificity was 62.5%, with a PPV of 16.8% and NPV of 94.1%.

B

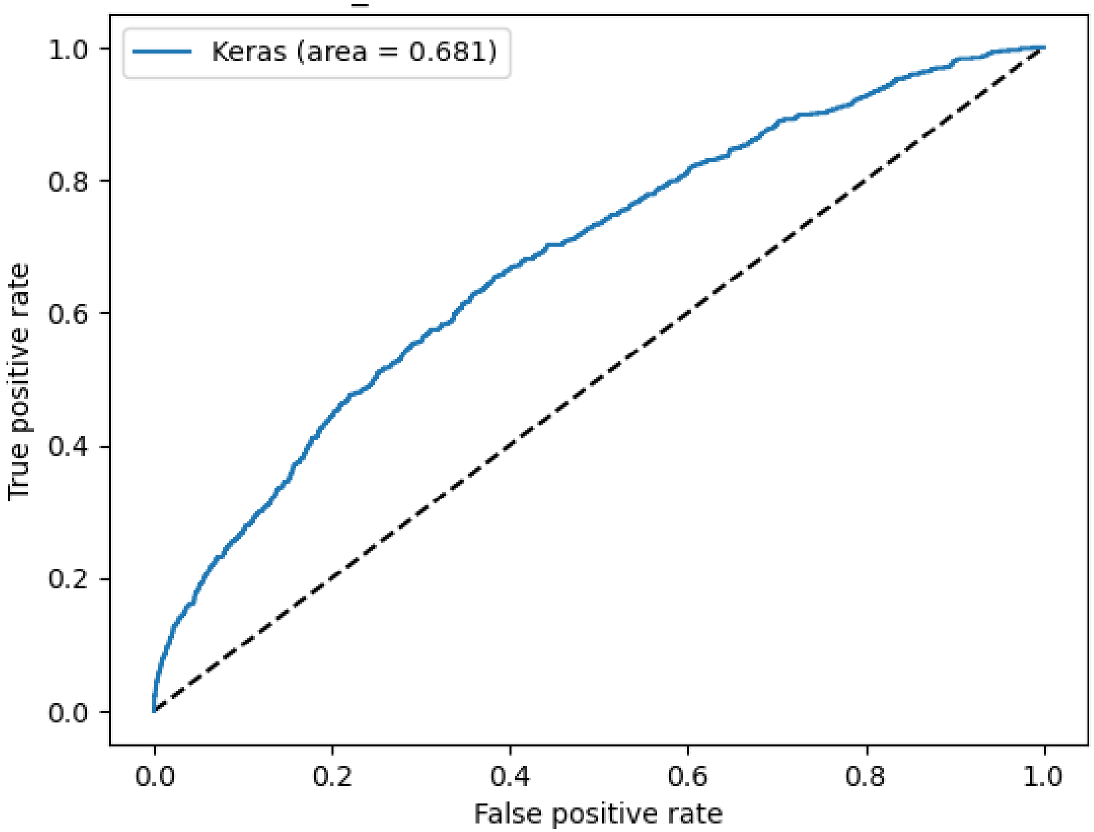

AUROC of 0.68, 95% CI 0.66-0.70. The sensitivity was 62.0% and the specificity was 61.5%, with a PPV of 17.5% and NPV of 93.5%.

Figure S6. Receiver Operating Characteristics Curve and Resulting Area Under the Curve for patients with Acute Pulmonary Embolism (red line) and Subgroup of Patients with Saddle Pulmonary Embolism (SADPE) or Right Ventricle Strain Pulmonary Embolism (RVSPE) using AI-DNN developed from the cohort of SADPE or RVSPE patients (blue line).

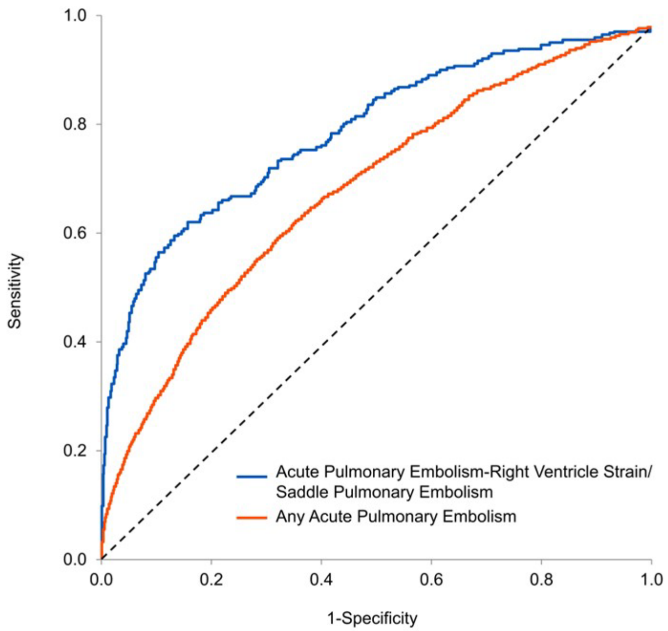

AUROC of a subgroup of patients with SADPE or RVSPE was 0.80 (95% CI 0.77-0.84). The sensitivity was 68.4% and specificity was 74.8%, with PPV of 4.1% and NPV of 99.3%.

Figure S7. Receiver Operating Characteristics Curve and Resulting Area Under the Curve for Patients with Saddle or Right Ventricle Strain Pulmonary Embolism diagnosed between 2010 and 2020 (last 10 years; panel A) and between 2015 and 2020 (last 5 years; panel B).

A

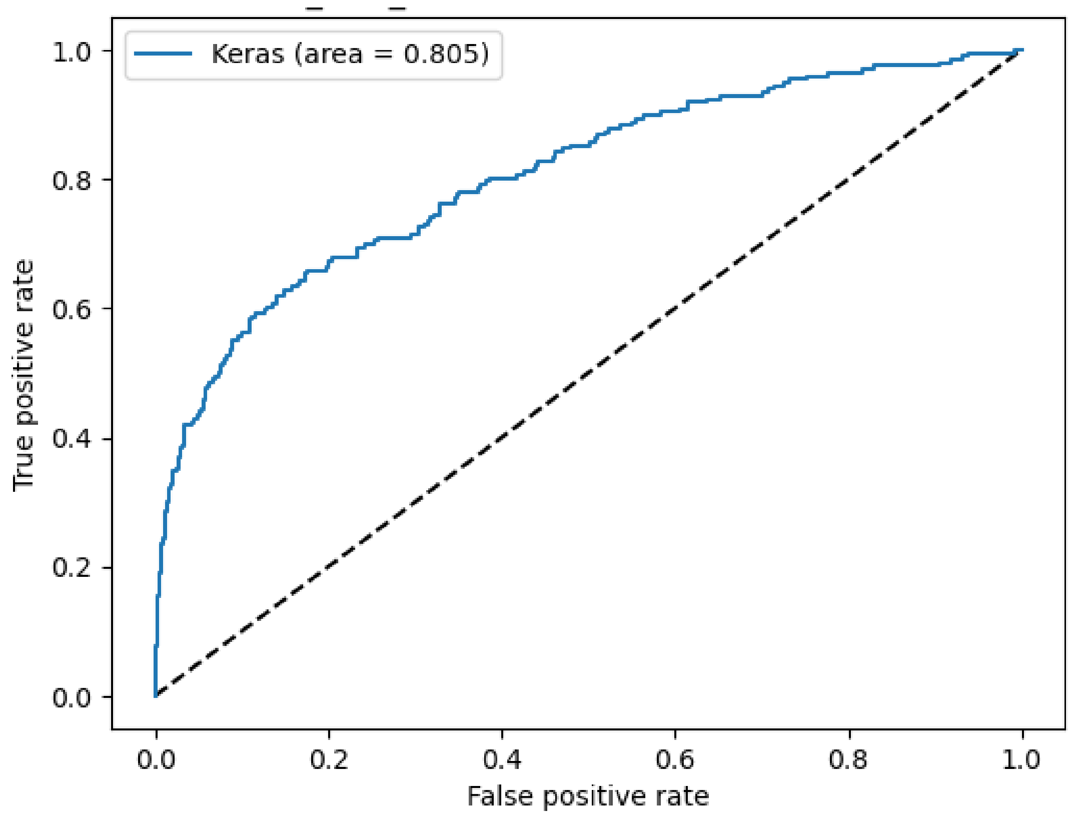

AUROC of 0.84, 95% CI 0.81-0.86 . The sensitivity was 82.6% and the specificity was 62.5%, with a PPV of 4.4% and NPV of 99.4%.

B

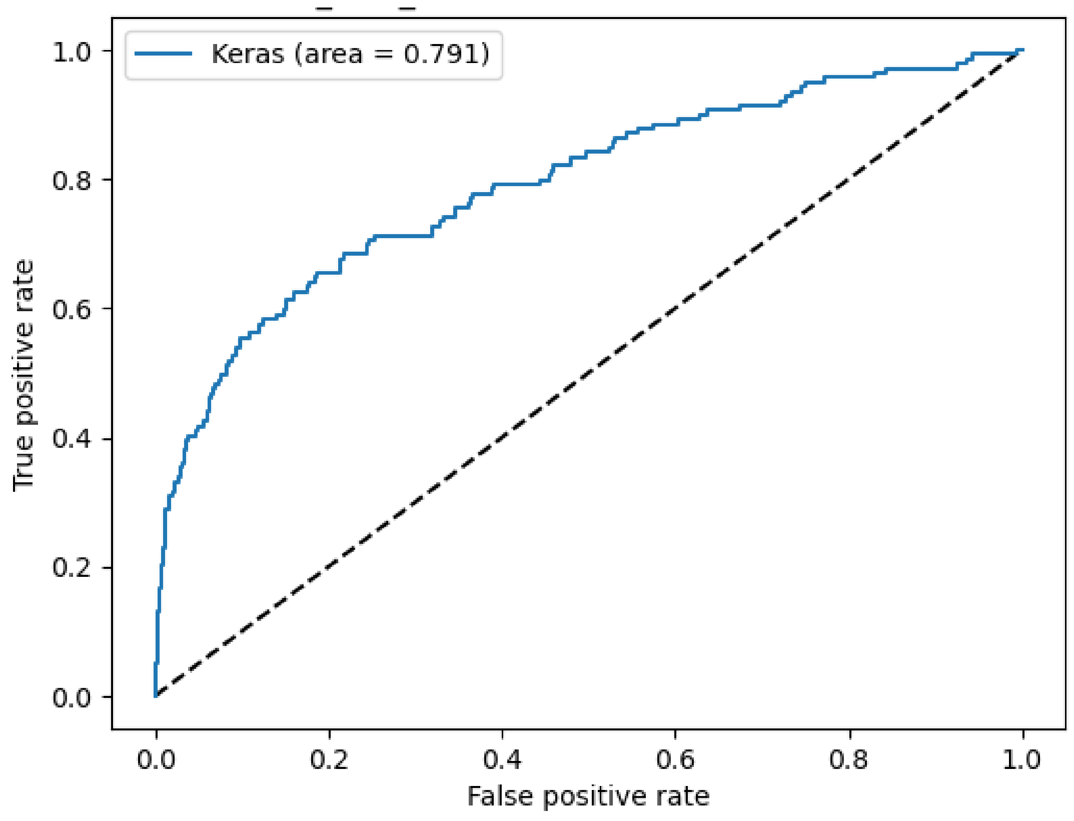

AUROC of 0.83, 95% CI 0.79-0.86. The sensitivity was 82.8% and the specificity was 61.5%, with a PPV of 4.9% and NPV of 99.3%.

Supplemental Table. The comparison of the sensitivity, specificity, positive predictive value (PPV), negative predictive values (NPV) and the receiver operating characteristics curve (AUROC) of two prior (27,28) and current study data for the whole cohort of patients with pulmonary embolism (PE), PE with right ventricle strain (RVSPE), saddle PE (SADPE) diagnosed by computed tomography pulmonary angiography (CTPA), that use machine learning models analyzing an ECG waveform only or combining with clinical data (fusion model, ref 27) . In addition, the same performance data for the electrocardiography models for PE diagnosis assessed in Su XF et al. study (ref 19) are provided.

Study	Number of Patients total	Number of Patients with PE(+)/PE(-)	PE Types	ECG Timing	Sensitivity (%)	Specificity (%)	PPV	NPV	AUROC
Outcome of machine learning models analyzing ECG waveform for PE diagnosis									
**Current study, whole cohort**	**79,894**	**7,423/72,471**	**All Acute**	**within 6 hrs of CTPA**	**63.50**	**64.70**	**15.60**	**94.50**	**0.6995**
**Current study, SADPE or RVSPE**	**73,609**	**1,138/72,471**	**SADPE or RVSPE**	**within 6 hrs of CTPA**	**80.77**	**64.67**	**3.54**	**99.52**	**0.8370**
Somani et al (27) ECG waveform only	25,099	3,397/23,793	Excluded subsegmental PE	within 24 hrs of CTPA					0.5900
Somani et al (27) ECG & clinical data	25,009	3,397/23,793	Excluded subsegmental PE	within 24 hrs of CTPA	100.0	18.0	26.0	100.0	0.8100
Silva et al (28) ECG waveform only	1,014	38/65*	Any PE**	unclear	50.0	100.0	100.0	77.38	0.7500
Electrocardiography models for PE diagnosis									
SPHH-ECG, Su XF (19)	327	327/331	All Acute PE, unclear if subsegmental excluded	48 hrs from the onset of patient symptoms	79.08	79.76	79.32	79.52	0.8741
Daniels ECG score (19)	327	327/331	All Acute, PE unclear if subsegmental excluded	48 hrs from the onset of patient symptoms	25.99	95.17	84.16	56.55	0.7528

*Only a number of positive and negative cases used for validation cohort was provided.

** Only patients with a D-dimer measurement obtained within 12 hours before CTPA were included
